# Breath therapy for patients with chronic nonspecific low back pain: A protocol of systematic review and meta-analysis

**DOI:** 10.1097/MD.0000000000021542

**Published:** 2020-07-31

**Authors:** Xu He, Chunjiang He, Xiaobing Luo, Huaimin Lu, Liang Wu, Duoduo Yu, Piming Gao, Wenqi Zhou, Hai Shen, Yaming Yu

**Affiliations:** a Sichuan Provincial Orthopedics Hospital; b Chengdu Sport University, China.

**Keywords:** breath therapy, chronic nonspecific low back pain, effectiveness, meta-analysis, protocol, safety, systematic review

## Abstract

**Background:**

Chronic nonspecific low back pain (CNLBP) has become a major global public health problem. Its high incidence rate and high disability rate are so damaging both to individuals and communities. At present, many countries’ clinical guidelines recommend exercise therapy. Breath therapy is one of the exercise therapies, playing an important role in exercise therapy. Some studies have shown that breath therapy has a considerable therapeutic effect on low back pain, but there is no specific conclusion. The aim of our study is to answer the question: if breath therapy is effective and safe for CNLBP?

**Methods:**

The following databases will be searched: English databases (including Web of Science, the Cochrane Library (Central), EMBASE, MEDLINE, Allied and Alternative Medicine) and Chinese databases (including Chinese National Knowledge Infrastructure, Wanfang data and Chinese Scientific Journals Database [VIP]). The literature search will be constructed around search terms for breath therapy, search terms for chronic nonspecific low back pain and search terms for randomized controlled trials. The primary outcomes were related to duration, intensity, attack frequency of pain, and the secondary outcomes were related to physical function, quality of life, and adverse events related to interventions. Endnote software 9.1 will be applied in selecting study, Review Manager software 5.3 will be applied in analyzing and synthesizing.

**Results:**

The results will provide evidence to judge whether breath therapy is effective and safe for CNLBP.

**Conclusion:**

Our research will provide reliable evidence of breath therapy for CNLBP.

**Registration:**

International Prospective Register of Systematic Reviews (PROSPERO) CRD42020156340.

## 1 Introduction

Low back pain was known as a symptom rather than a disease. It was defined by the location of the pain. When pain occurred between the lower ribs margins and the buttock creases, it was called low back pain.^[1]^ It was reported that the prevalence of low back pain was 23%^[2]^ with higher prevalence in females.^[3]^ The incidence rate of low back pain in adolescents was similar to that in adults.^[4]^ It was surveyed in 2016, low back pain was the leading cause of disability in the whole year globally, affecting 57.6 million people.^[5]^ Low back pain had a bad influence on personal income, work ability and mental health, which damaged patients’ quality of life and brought heavy economic burden to family and society.^[1]^ In the United Kingdom, it was found that the national economic burden of low back pain was similar to that of high cost diseases such as cardiovascular disease, cancer and autoimmune disease.^[6]^

Low back pain lasting for more than 12 weeks was defined as chronic^[7–9]^ and less than 12 weeks was defined as acute.^[10]^ The results of relevant researches showed that 2 thirds of low back pain patients had pain for more than 12 weeks, 65% for more than 12 months,^[11]^ 1 year recurrence rate was between 24% and 80%,^[12]^ which suggested that chronic low back pain was the majority of the disease. Most chronic low back pain cannot be determined by pathoanatomical diagnosis, which was called chronic nonspecific low back pain (CNLBP), and the key point of treatment was to relieve symptoms and improve its prognosis,^[1,13]^ so as to improve the quality of life of patients. Recently, the common treatment of CNLBP were nonsteroidal antiinflammatory drugs (NSAIDs), opioid, spinal injection, surgery, and radiofrequency denervation all over the world,^[8]^ without any significant improvement in either prognosis or disability rates of the patients though.^[14]^ Besides, long term use of drugs carried some risk of adverse effects.^[15–17]^ At present, clinical guidelines in some countries have shifted the focus from drugs and surgical treatment to enhancing function.^[8]^ It was reported that exercise therapy can reduce the recurrence rate by 35%^[18]^ and was recommended in the clinical guidelines of many countries.^[8,13]^ Breath therapy was one of the exercise therapies, playing an important role in treating CNLBP. Respiratory muscles of patients with low back pain were more prone to fatigue, the decrease of respiratory muscle function will lead to the decrease of oxygenation and blood volume of back muscle.^[19]^ Some studies have shown that the strength of respiratory muscles was increased, and the degree of low back pain was reduced.^[20]^ It was observed in our clinic, breath therapy was effective and safe for CNLBP. However, there was no authoritative specific evidence nowadays. Hence, the aim of our study is to systematically synthesize all randomized controlled trials (RCTs) of breath therapy for patients with CNLBP to provide evidence for the clinical practice.

## 2 Methods

### 2.1 Study registration

The protocol of our study has been registered with International Prospective Register of Systematic Reviews (PROSPERO) (registration number: CRD42020156340). The protocol is reported strictly according to the Preferred Reporting Items for Systematic Reviews and Meta-Analyses Protocols (PRISMA-P) guidelines.

### 2.2 Eligibility criteria

#### 2.2.1 Type of study

We will include the RCTs of breath therapy for patients with CNLBP.

#### 2.2.2 Type of participant

The study involved participants who had been diagnosed with CNLBP.

CLBP is usually defined as pain, or uncomfortable below the costal margin and above the inferior gluteal folds for no less than 12 weeks or more,^[7–9]^ with or without leg pain.^[21]^ The diagnosis of CNLBP needs to be further exclusion of diseases with definite pathoanatomical causes, such as radicular syndrome, cauda equina syndrome, spinal stenosis, cancer, compression fracture, spinal infection, osteoporosis, ankylosing spondylitis, and etc.^[22]^ There is no restriction of age, gender, or race.

#### 2.2.3 Type of intervention

Our research will include studies that took breath therapy as the main treatment in the intervention group, such as pranayama, qigong, breathing control, etc. Meanwhile, the control group using nonbreath therapy (pharmacological treatments, placebo) or waiting-list.

#### 2.2.4 Types of outcome measurements

The primary outcome will be set to evaluate pain, including duration, intensity, and attack frequency. The secondary outcomes will be set to evaluate physical function, quality of life, and adverse events related to interventions.

#### 2.2.5 Exclusion criteria

(1) Participants without clear diagnosis;
(2) Breath therapy was not set as primary treatment in the intervention group;
(3) Data could not be extracted.
(4) Data duplication;
(5) The studies where full text was unavailable.

### 2.3 Search methods for identification of studies

#### 2.3.1 Electronic data sources

The following 10 electronic databases from inception to July 2020 will be searched: Web of Science, the Cochrane Library, EMBASE, MEDLINE, ISI Web of Knowledge, Allied and Alternative Medicine, Chinese National Knowledge Infrastructure (CNKI), Wanfang data and Chinese Scientific Journals Database (VIP).

#### 2.3.2 Other resources

Relevant references will be reviewed and screened. In addition, we will search the following registration website of the clinical trial: WHO ICTRP, http://www.chictr.org.cn, http://www.ClinicalTrial.gov, and ISRCTN Register. Moreover, the relevant grey literature from the Health Management Information Database (HMIC), Open SIGLE Database, and the National Technical Information Service (NTIS) will be searched. Experts in the field will be consulted for relevant studies.

### 2.4 Search strategy

Subject words and text words will be combined for the search strategy. The search terms will be expanded around: breath therapy, chronic nonspecific low back pain, and randomized controlled trial. It will not be restricted with publication dates and languages. Use MEDLINE as an example, the specific searching strategy, as stated in Table 1. The searching strategy will be modified by the varied characteristic of the different databases.

**Table 1:**
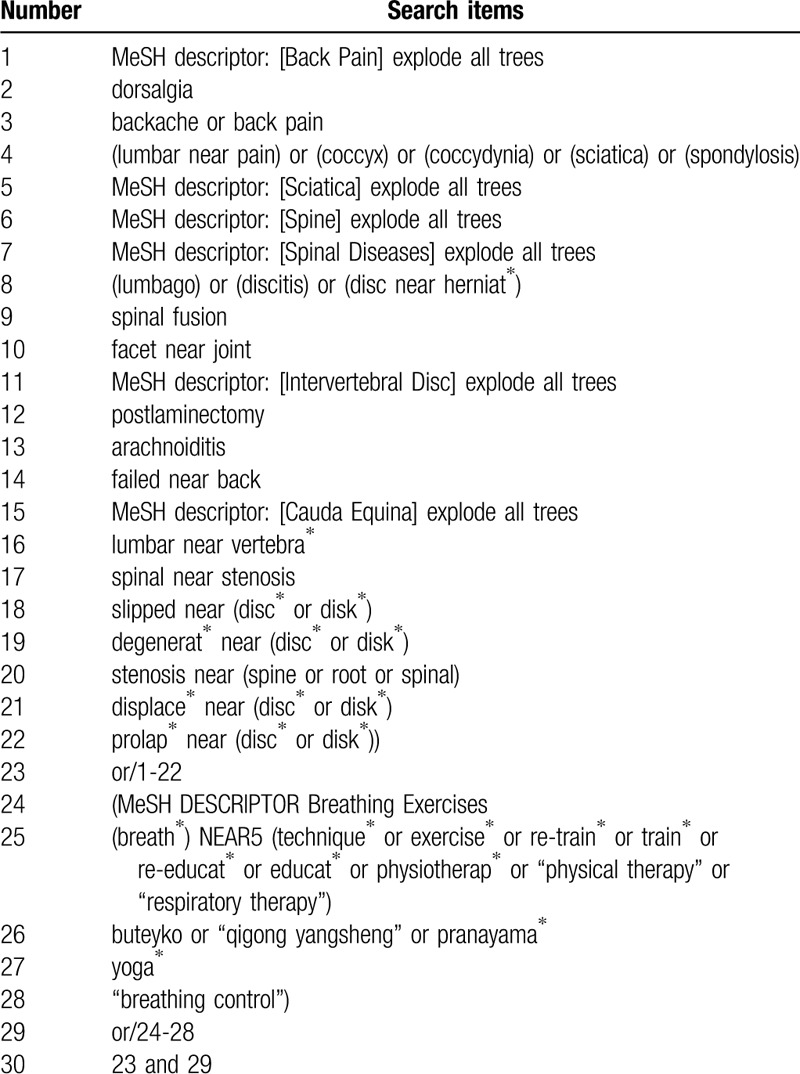
Search strategy for the MEDLINE database.

### 2.5 Data collection

#### 2.5.1 Selection of studies

**Figure 1:**
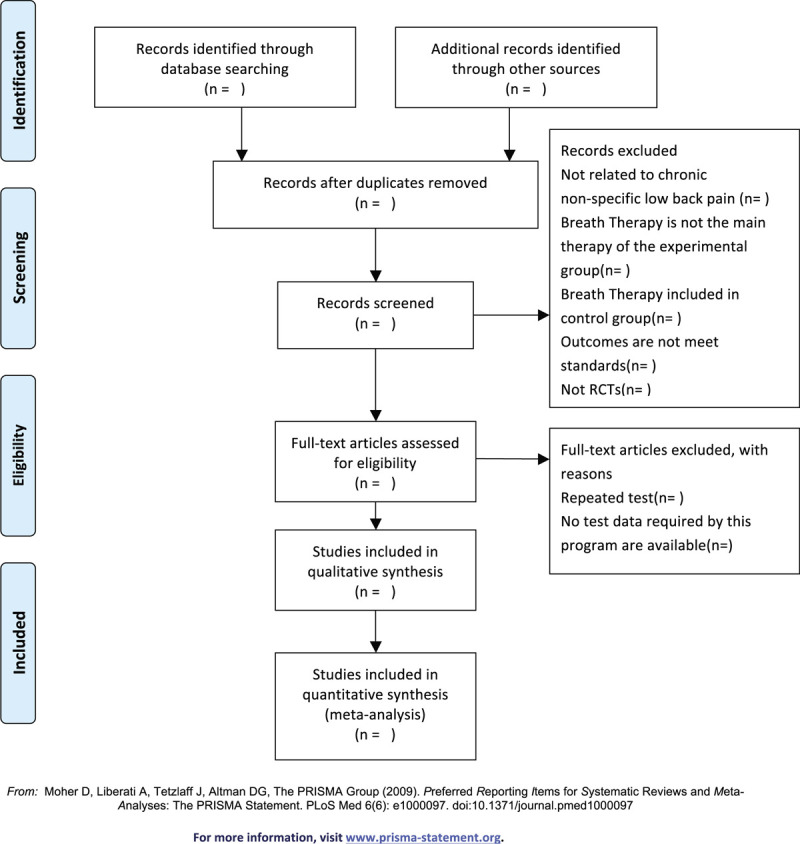
The Preferred Reporting Items for Systematic Reviews and Meta-analyses Protocols flow diagram of the study selection process.

The retrieved studies will be imported in Endnote software 9.1 to remove duplicates. Two researchers (XBL and HML) will screen the titles and abstracts independently according to the preestablished inclusion and exclusion criteria. After that, the full text will be screened as a second filtration. Two researchers will crosscheck the included studies, and the third researcher (YMY) will be involved if disagreements occur. The detailed screening process will be shown in the following Preferred Reporting Items for Systematic Reviews and Meta-Analyses Protocols (PRISMA-P) flow diagram (Fig. 1).

#### 2.5.2 Data extraction and management

The other 2 researchers (PMG and WQZ) will extract data independently to fill out the predesigned form. The information includes author, country, publication year, methodological quality, characteristics of participants, the details of intervention and comparisons, outcomes, the specific data, results, conclusions, follow-up, adverse events, conflicts of interest, sources of funds, and ethical approval. The extracted data will be crosschecked by the 2 researchers. A third researcher (CJH) will be involved if a disagreement occurs. The authors of the studies included will be contacted for further information when necessary.

#### 2.5.3 Assessment of risk of bias in included studies

According to the guidance from the Cochrane Handbook of Systematic Reviews of Interventions,^[23]^ 2 researchers (XH and DDY) will evaluate the risk of bias of the included RCTs independently. We will evaluate from the following 6 parts: selection, performance, attrition, detection, reporting, and other sources of bias. We will rate the risk of bias into 3 levels: when meets none of the criteria, it will be regarded as high; when meets all criteria, it will be regarded as low; when study without sufficient information to determine, it will be regarded as unclear. After the assessment, it will be crosschecked by 2 researchers. The third researcher (HS) will be involved if a disagreement occurs.

### 2.6 Data synthesis

Review Manager software (RevMan5.3) will be used to conduct all data analyses if it is possible to perform a meta-analysis. Data synthesis will be performed with a random-effects model if significant statistical heterogeneity is detected. Otherwise, the data will be processed with a fixed-effects model. Furthermore, the descriptive analysis will be conducted if there is significant statistical heterogeneity.

#### 2.6.1 Measures of treatment effect

For continuous outcomes (the change in duration, intensity, and attack frequency of pain after treatment, the change in physical function after treatment, and the change in quality of life after treatment), we will use mean difference to evaluate the extracted data. For dichotomous outcomes (the cure rate, the total effective rate and the recurrence rate which were set to evaluate the change of pain and adverse events related to interventions), we will analyze the rate ratio. The confidence intervals will be set to 95% for both continuous outcomes and dichotomous outcomes.

#### 2.6.2 Management of missing data

The related corresponding author will be contacted if there are insufficient or missing data. If accurate data is still unavailable after contacting the corresponding author, these studies will be excluded.

#### 2.6.3 Assessment of heterogeneity

We will conduct the qualitative analysis by comparing the characteristics of included researches and quantitative analysis by using the *I*^2^ test and the χ^2^ test to assess the heterogeneity. If the values of *I*^2^ more than 50%, the significant heterogeneity will be thought to exist.

#### 2.6.4 Assessment of reporting biases

If the quantity of the included RCTs was no less than 10, funnel plots will be selected to evaluate the potential publication bias.

#### 2.6.5 Subgroup analysis

According to different kinds of breath therapy applied, different interventions of the control group, and different time points for evaluating outcomes after treatment, subgroup analysis will be performed.

#### 2.6.6 Sensitivity analysis

Based on the risk of bias, insufficient data, and sample size, we will perform a sensitivity analysis to evaluate the robustness if significant statistical heterogeneity existed.

### 2.7 Grading the quality of evidence

According to the Grading of Recommendations Assessment, Development, and Evaluation (GRADE),^[24]^ we will assess each outcome's quality of evidence from limitation of study design, inconsistency, indirectness, imprecision, and bias of publication, ranking the quality into 4 different levels: very low, low, moderate, and high).

### 2.8 Ethics and dissemination

There is no necessity to gain ethical approval considering our research has no connection with individual patient data. The results of our research will be reported in a peer-reviewed journal or relevant conferences and evaluate the implication of breath therapy for patients diagnosed with CNLBP.

## 3 Discussion

CNLBP occurs in high-income, middle-income and low-income countries.^[1]^ It has an impact on people of all ages and is the main disease burden in the world.^[13]^ CNLBP has no definite pathoanatomical diagnosis, and there was no consensus treatment recommendations.^[8]^ Breath therapy may be an effective treatment, which may be related to the improvement of respiratory muscle function.^[25]^ Our systematic review and meta-analysis will be conducted based on the existing RCTs to assess the effectiveness and safety of the breath therapy for CNLBP.

## Author contributions

The idea of this study was put forward by Xu He and Yaming Yu. The protocol was drafted by Xu He and Chunjiang He. The whole process was supervised by Yaming Yu, Hai Shen. The manuscript was revised by all authors. The final version was approved by all authors.

**Conceptualization:** Xu He, Yaming Yu. Methodology: Xu He, Xiaobing Luo, Huaimin Lu, Duoduo Yu, Piming Gao, Wenqi Zhou. Supervision: Yaming Yu, Hai Shen. Writing – original draft: Xu He, Chunjiang He.

**Writing – review & editing:** Xu He, Liang Wu.
